# An Account of a Contagious Disorder, Called the Venom, Which Has Prevailed Lately Amongst the Horned Cattle in Friesland

**Published:** 1783

**Authors:** 


### SECTION II.

### Essays and Observations.

I. *An Account of a contagious disorder, called the Venom, which has prevailed lately amongst the horned cattle in Friesland. Communicated in a letter to* Samuel Foart Simmons, M. D. F. R. S. *By* Petrus Camper, M. D. F. R. S. *honorary professor of physic, anatomy, and surgery at Amsterdam, fellow of the Royal College of physicians and Royal Society of Edinburgh, of the Imperial Academy of Petersburgh, and of the Royal Medical Society at Paris, &c.*

A Great number of black cattle of all ages have died suddenly in Friesland in the neighbourhood of Sneek and Ylst, without any previous previous ſymptom but that of giving no milk a few hours before their death, as they continued to eat, drink and ruminate to the laſt. In general, however, the beaſts affected with this diſorder lived ſeveral days, and had very large tumours in the cellular membrane, about the head and neck, in the axilla, and ſometimes, though rarely, in the groin. Theſe tumours, which were often as large as a man's head, were very hard and elaſtic, with a dry horny ſkin at the part which was moſt prominent. In ſome, this ſwelling entirely diſappeared, while in others the dry piece of ſkin ſeparated and left a large ulcer, which healed ſlowly. Some were ſoon relieved; others required many days, and even weeks, before they were entirely cured, and many, as I have already obſerved, died ſuddenly, or after a relapſe. The dung was in the greater number natural, but in ſome it was of a blackiſh colour, or at leaſt darker than uſual.

The city of Sneek is about twelve Engliſh miles from my ſeat, and I paſſed ſeveral days there in examining the diſorder, and diſſecting the cattle that died of it. This latter part of my inquiry, however, was not to be carried on without danger of being poiſoned by the blood, fleſh, and ſkin, &c. of the animal.

The difeafe has gotten the name of *venom*, or *venenum*, from this circumftance, that the people who handle the hide or the flefh, are often poifoned, as it were, in the hands, efpecially when they have any fcratch or wound there to favour abforption, and fometimes without any fuch previous hurt. In a few hours an inflammation takes place, and, if not fpeedily prevented by fcarifications and fuitable remedies, terminates in a gangrene which fometimes fpreads to the arm, and in fome few inftances has occafioned the death of the patient. It is worthy of obfervation however, that after the flefh of animals, who have died of this difeafe, has been boiled, the poor eat it without any bad confequence, but they carefully avoid the fteam of the meat.

On the 6th of September laft I opened a cow that had died the night before at Ylft, which is not far from Sneek. In my way I vifited an old man whofe hands were fo much affected by the venom, that I was almoft deterred from attempting the intended diffection. I ufed the precaution, however, of greafing my hands with pomatum, and by frequently wafhing them and renewing the ointment, I prevented the poifonous juices from acting, fo that altho' I fepa-

I ſeparated the viſcera, &c. with my hands, I got not the leaſt hurt.

The eyes, tongue, and throat of the cow were found. The udders were without milk, but in other reſpects healthy, and there was no where any appearance of tumour. But on opening the abdomen we found the omentum entirely mortified, with a yellowiſh ichor within its cavity, and between the inteſtines were obſerved thick purulent coagulated membranes, ſimilar to thoſe appearances which are found in the human body, where death has been occaſioned by an inflammation of the bowels.

Neither of the ſtomachs were affected, but the Duodenum, Jejunum, and Ileum were inflamed and mortified, as likewiſe the colon in ſome places. The gall bladder, which was uncommonly diſtended, being larger than even the urinary bladder of a cow uſually is, was filled with air, and with a very thin bile. The uterus was a little inflamed with gangrenous ſpots. The calf had been dead ſome time, but the cow continued to give her ordinary portion of milk till the day before ſhe died. The liver ſeemed to be pretty ſound, but its lymphatics were viſible and much enlarged. The ſpleen was in a gana gangrenous ſtate, and there was emphyſema between the duplicature of the peritoneum, where it forms the meſentery.

The lungs were in a natural ſtate, but the glandulæ cordis and the thymus were much inflamed. The heart itſelf was in a good condition.

I examined ſeveral other beaſts that were affected with the ſame diſorder, which was evidently of the putrid kind. The pulſe was quick and low, as it is in all putrid fevers, and I had reaſon to ſuſpect that thoſe died very ſuddenly, whoſe blood was much affected by the putrid matter, and on the contrary, that others ſoon recovered whoſe blood had a better diſpoſition. The tumours were neither a good nor a bad ſign, for many died and as many recovered with and without any ſuch ſwelling. The peaſants told me, that an old mare died of the venom, but upon opening her abdomen and thorax after death, I found nothing analagous to the diſorder I have been deſcribing.

The diſeaſe abated much towards the latter end of September, and the contagion is now totally over. I could find no account of this diſeaſe in books, till I conſulted the famous

Dr.

Dr. Pallas's *Northern Magazine** (Vol. I. B. I. § 4, p. 113) in which Dr. Jos. James Lerche has given a description of a contagious disorder that made great havoc, after a hot dry summer in 1756, amongst the horned cattle in Livonia and Finland, and which spread even as far as Moscow. He informs us, that the cows were attacked with large tumours in the neck, breast, belly, and pudenda, and commonly died in two or three days. He adds, that horses and hogs were likewise susceptible of the contagion which generally carried them off in a day or two, and that it also proved fatal to a number of the human species; but upon inquiry he found that the latter died of a mortification of the hands, &c. occasioned by an absorption of the venom. A similar plague was observed in those countries in the year 1764.

I flatter myself the description I have given of this disease, though short, will be sufficient to give you an idea of its nature, and to enable you and your medical friends to compare it with the symptoms of the disorder which was observed lately in England: for I have reason to

* Nordische Beytrage.

believe believe that the latter was of the same species, and of course different from that described by Dr. Layard and others, which, by the bye, still prevails in this country. It may not be improper to add, that calves borne by cows that have passed through the latter distemper are inoculated * here with great success.

I am now growing old, but I have not lost my public spirit and zeal for useful improvements, so that I shall be very much obliged to you for any information you can furnish me with relative to the disorder I have mentioned to you. I am with the greatest respect and sincerity,

Dear Doctor,

Yours, &c.

*Klein Lancum,*

*Oct.* 21, 1783.

* See our 3d Volume, page 356.

